# Describing global pediatric RSV disease at intensive care units in GAVI-eligible countries using molecular point-of-care diagnostics: the RSV GOLD-III study protocol

**DOI:** 10.1186/s12879-021-06544-3

**Published:** 2021-08-23

**Authors:** Yvette N. Löwensteyn, Natalie I. Mazur, Harish Nair, Joukje E. Willemsen, Ghislaine van Thiel, Louis Bont, the RSV GOLD III—ICU Network study group, Maria Ahuoiza Garba, Fatima Jumai Giwa, Mohammad Hafiz Rasooly, Aminullah Shirpoor, Merwais Azizyar, Lamin Makalo, Ousman Nyan, Ali Mohamed, Khalid Osman, Ram Hari Chapagain, Krishna Prasad Bista, Arun Kumar Sharma, Prabina Shrestha, Bamenla Goka, Kwabena Osman, Evangeline Obodai, Henshaw Mandi, Lucas Esuh Esong, Charlotte Ekoube Eposse, Valéria Muando, Tufária Mussá, Yasser Habresh Said, Aika Abia Shoo, Vanessa Jaelle Dor, Jacqueline Gautier, Linda Abicher

**Affiliations:** 1 Division of Infectious Diseases, Department of Paediatrics, University Medical Centre Utrecht, Utrecht, The Netherlands; 2 Centre for Global Health, Usher Institute, Edinburgh Medical School, University of Edinburgh, Edinburgh, UK; 3 Respiratory Syncytial Virus Network (ReSViNET) Foundation, Zeist, The Netherlands; 4 Julius Center for Health Sciences and Primary Care, Medical Humanities Department, University Medical Center Utrecht, Utrecht, The Netherlands

**Keywords:** Respiratory syncytial virus, Children, Pediatric intensive care unit, Study design, Lower-middle-income countries, Burden, Awareness

## Abstract

**Background:**

Respiratory syncytial virus (RSV) infection is an important cause of hospitalization and death in young children. The majority of deaths (99%) occur in low- and lower-middle-income countries (LMICs). Vaccines against RSV infection are underway. To obtain access to RSV interventions, LMICs depend on support from Gavi, the Vaccine Alliance. To identify future vaccine target populations, information on children with severe RSV infection is required. However, there is a lack of individual patient-level clinical data on instances of life-threatening RSV infection in LMICs. The RSV GOLD III—ICU Network study aims to describe clinical, demographic and socioeconomic characteristics of children with life-threatening RSV infection in Gavi-eligible countries.

**Methods:**

The RSV GOLD-III—ICU Network study is an international, prospective, observational multicenter study and will be conducted in 10 Gavi-eligible countries at pediatric intensive care units and high-dependency units (PICUs/HDUs) during local viral respiratory seasons for 2 years. Children younger than 2 years of age with respiratory symptoms fulfilling the World Health Organization (WHO) “extended severe acute respiratory infection (SARI)” case definition will be tested for RSV using a molecular point-of-care (POC) diagnostic device. Patient characteristics will be collected through a questionnaire. Mortality rates of children admitted to the PICU and/or HDU will be calculated.

**Discussion:**

This multicenter descriptive study will provide a better understanding of the characteristics and mortality rates of children younger than 2 years with RSV infection admitted to the PICU/HDU in LMICs. These results will contribute to knowledge on global disease burden and awareness of RSV and will directly guide decision makers in their efforts to implement future RSV prevention strategies.

*Trial registration number:* NL9519, May 27, 2021

**Supplementary Information:**

The online version contains supplementary material available at 10.1186/s12879-021-06544-3.

## Background

Respiratory syncytial virus (RSV) infection is an important cause of hospitalization and mortality due to lower respiratory tract infection (LRTI) in children under 5 years of age worldwide [1]. Annual RSV-related hospital admissions and in-hospital deaths in this age group have been estimated to be 3.2 million and 59,600, respectively, while overall annual RSV-related mortality including community deaths could be as high as 118,200 [1]. The majority of deaths (99%) occur in low- and lower-middle-income countries (LMICs) due to lack of access to healthcare and poor quality of care in health facilities [1], and children under 2 years of age are disproportionally affected [2, 3]. As the Haemophilus influenza type b and pneumococcal conjugate vaccines are introduced and scaled up in LMICs, the global burden of child pneumonia attributable to bacterial causes has decreased and the proportional contribution of viral pathogens has increased. RSV now remains one of the major pathogens that needs to be tackled in order to achieve sustainable development goal 3.2—end preventable deaths of newborns and children under 5 years of age by 2030.

Currently there is no immunization available against RSV, although several vaccine and monoclonal antibody candidates are under clinical development [4]. The most advanced maternal vaccine candidate has completed a phase 3 trial but did not meet the primary endpoint [5]. A new extended half-life monoclonal antibody developed by SanofiPasteur / MedImmune, nirsevimab (previously MEDI8897), has met the primary endpoint of reducing RSV LRTI in healthy infants in a recent phase III trial [6].

Gavi, the Vaccine Alliance (previously: Global Alliance for Vaccines and Immunizations), is an international organization that invests in vaccines to protect children’s lives and health in LMICs. Every 5 years, Gavi develops a new vaccine investment strategy (VIS) to prioritize new and under-used vaccines and to make these available to LMICs through the Gavi vaccine support programme. RSV interventions, including both maternal vaccine and monoclonal antibodies, were considered as one of the six prioritized vaccine programmes as part of Gavi VIS for the 2021–2025 funding period [7]. It is anticipated that future RSV vaccines or monoclonal antibodies will be most efficacious in targeting severe disease leading to poor outcome (e.g. oxygen supplementation, ICU admission, and death).

The majority of Gavi-eligible countries have sparse or no individual patient-level data to make decisions on target populations for RSV interventions when these become available in the next 5–10 years. These data will be important for cost-effectiveness analyses of potential RSV interventions to assist policy makers in making decisions related to resource allocation for RSV interventions [8]. Patient-data will also contribute to local disease awareness. Defining burden in terms of RSV incidence and case-fatality ratios in Gavi-eligible countries has been challenging due to insufficient diagnostic capabilities for RSV surveillance [9]. Interviews with stakeholders revealed that RSV prevention received low priority at national and government level due to lack of information about disease and disease burden, and some respondents suggested that RSV diagnostics would help to improve value proposition [10].

This study aims to obtain individual patient-level data from children who have been admitted with severe RSV infection at the (pediatric) intensive care unit (ICU) or high-dependency unit (HDU) in Gavi-eligible countries through implementation of RSV point-of-care testing to pave the way for future vaccine introduction.

## Methods/design

### Study design and study site selection

The RSV GOLD III study is a prospective, observational, multi-centre study and will be conducted at 11 sites in 10 Gavi-eligible countries. The study was initiated at the first study sites in April, 2021. The total duration of the study is 2 years for each participating study site. To select study sites, we sent out an open invitation for collaboration to researchers and physicians from various LMICs from the existing RSV GOLD network. Before the start of the study, we collected information about potential study sites through email correspondence and teleconferences. The minimum collected information included but was not limited to the location and catchment area of the hospital, the hospital staff to be in charge of performing the study, logistics of the hospital (languages spoken, freezer availability, respiratory seasonality), pediatric ward, pediatric or neonatal ICU, and (pediatric) HDU availability and number of beds, the annual number of respiratory illness-related admissions, and mortality rates. If admission data were not available, estimations were made by the study team based on number of beds and information from local collaborators. We selected study sites based on a high expected number of RSV inclusions, quality of the communication and engagement of local collaborators.

### Study sites

The study will be conducted in the following LMICs: Afghanistan, Cameroon, Ghana, Haiti, Mozambique, Nepal (2 hospitals), Nigeria, Sudan, Tanzania, and The Gambia. The study will also be conducted at 2 sites in the Netherlands to allow for a comparison of patient characteristics with patients from a high-income country (HIC). Table 1 provides the characteristics of the participating LMIC study sites.

**Table 1:** Characteristics of the participating RSV GOLD III - ICU Network study sites in low- and lower-middle-income countries

City, Country	Hospital	Number of PICU Beds	Number of HDU Beds	Number of NICU beds	Estimated annual number of children < 2 years admitted to PICU / HDU / NICU with severe acute respiratory infection	Respiratory season
Zaria, Nigeria	Ahmadu Bello University Teaching Hospital	NA	32*	8	155	April–November
Mazar-e-Sharif, Afghanistan	Balkh regional hospital	35	NA	8	650	October–March
Banjul, The Gambia	Edward Francis Small Teaching Hospital	NA	20	37	100	October-May
Khartoum, Sudan	Jafar Ibn Auf Specialized Hospital for Children	8	9	16	100	December-May
Kathmandu, Nepal	Kanti Children’s Hospital	12	8	16	130	July-March
Kathmandu, Nepal	Tribhuvan University Teaching Hospital	4	6	8	32	July-March
Douala, Cameroon	Laquintinie Hospital Douala	20	NA	NA	180	September-January April-June
Accra, Ghana	Korle Bu Teaching Hospital	6	18	50**	70	June-November
Maputo, Mozambique	Maputo Central Hospital	21	NA	70**	85	March-August
Dar es Salaam, Tanzania	Muhimbili National Hospital	13	NA	19**	270	December-May
Port-au-Prince, Haiti	Saint-Damien Hospital	10	NA	16**	40	August-January

*PICU* pediatric intensive care unit, *HDU* high dependency unit, *NICU* neonatal intensive care unit, *NA* not available

*Emergency unit serves as HDU

**No recruitment

### Study objectives

#### Primary objective

To describe the clinical, demographic, and socioeconomic characteristics of RSV-positive children under 2 years of age who have been admitted with suspected RSV infection at ICUs or HDUs in Gavi-eligible countries.

#### Secondary objectives

1. To determine proportional RSV-related mortality in children under 2 years of age at participating ICUs or HDUs.
2. To compare clinical, demographic, and socioeconomic characteristics between children with fatal and non-fatal RSV infection.
3. To compare clinical, demographic, and socioeconomic characteristics between children with RSV infection from LMICs and HICs.
4. To describe RSV seasonality in the study locations.
5. To compare clinical, demographic, and socioeconomic characteristics between children with (fatal) RSV infection and children with (fatal) influenza infection.
6. To confirm the point-of-care (POC) RSV test using conventional or real-time RSV PCR.
7. To estimate the sensitivity of the World Health Organization (WHO) “extended severe acute respiratory infection (SARI)” case definition for hospital-based surveillance for severe RSV infection. [11]
8. To compare the burden of RSV infection and mortality rates between children who do and do not meet the WHO “extended SARI” case definition.

### Study participants

**Fig. 1:**
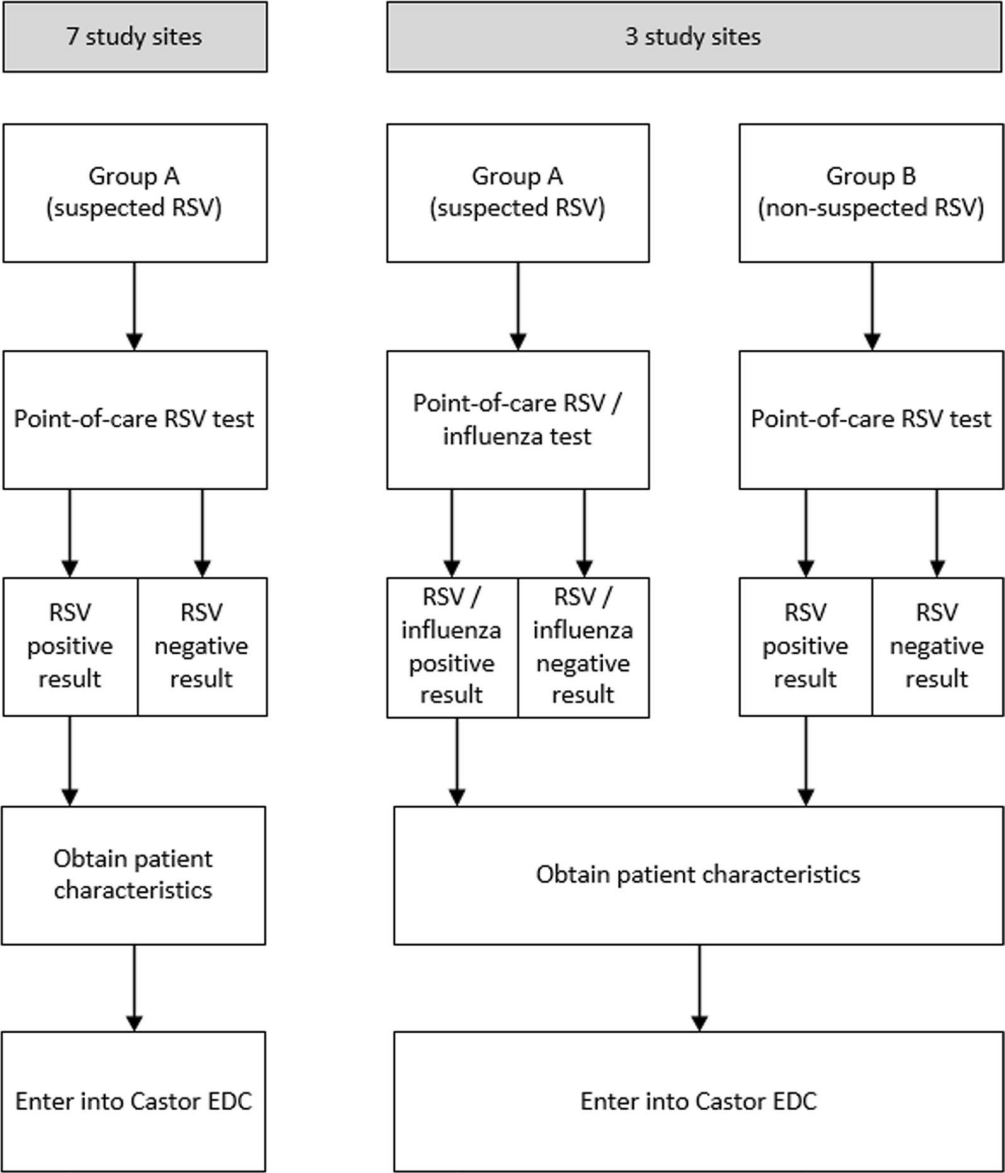
Schematic diagram of the study workflow. Group A: children < 2 years admitted to the PICU and/or HDU meeting the WHO “extended SARI” case definition (10 study sites). Group B: children < 2 years admitted to the PICU and/or HDU not meeting the WHO “extended SARI” case definition (3 study sites: Ghana, Mozambique, Nepal)

For this study, 2 different groups of children (A and B) are distinguished. For each group, a subject must meet all the eligibility criteria in order to participate (Table 2). Children from group A will be tested for RSV at all study sites and for influenza at 3 study sites (Ghana, Mozambique, Nepal) (Fig. 1):

**Table 2:** Eligibility criteria for RSV GOLD III—ICU Network Study

Inclusion criteria	
Group A	Group B
Age < 2 years at time of sampling; Admitted to PICU / HDU / NICU; Meeting WHO “Extended SARI” case definition: Severe (defined as requiring hospitalization); and Acute (defined as onset within the last 10 days); and Respiratory infection (defined as having cough or shortness of breath) In infants less than 6 months, additionally include those who present with: Apnea (temporary cessation of breathing from any cause); and/or Sepsis, defined as: Fever (37.5 °C or above) or hypothermia (less than 35.5 °C); and Shock (defined as lethargy, fast breathing, cold skin, prolonged capillary refill or fast weak pulse); and Seriously ill with no apparent cause Signed and dated written informed (deferred) consent obtained from the parent(s)/legal representative(s) of the subject, or in accordance with local regulations	Age < 2 years at time of sampling; Admitted to PICU / HDU / NICU; Signed and dated written informed (deferred) consent obtained from the parent(s)/legal representative(s) of the subject, or in accordance with local regulations

*PICU* pediatric intensive care unit, *HDU* high dependency unit, *NICU* neonatal intensive care unit, *WHO* world health organization, *SARI* severe acute respiratory infection

#### Group A. Children with suspected RSV disease (all study sites)

1. Children < 2 years of age at time of sampling;
2. Who are admitted to an ICU and/or HDU and meet the WHO “extended SARI” case definition;

Children who do not meet the WHO “extended SARI” case definition will be tested for RSV at 3 study sites (group B):

#### Group B. Children who are not suspected to have RSV disease (Ghana, Mozambique, Nepal)

1. Children < 2 years of age at time of sampling;
2. Who are admitted to an ICU and/or HDU and do not meet the WHO “extended SARI” case definition;

### WHO “extended SARI” RSV surveillance case definition

In this study, the global case definition developed by the WHO for hospital-based RSV surveillance is used to identify children hospitalized with suspected RSV infection. The RSV surveillance case definition was recently modified based on results from the WHO RSV surveillance pilot which showed that the use of an *extended* SARI case definition, not requiring fever to identify a suspect case, substantially increased the number of RSV infections detected [3].

In group A, subjects will be tested for RSV when they are admitted to the ICU meeting the WHO “extended SARI” case definition for hospital-based surveillance for severe RSV infection:

- Severe (defined as requiring hospitalization); and
- Acute (defined as onset within the last 10 days); and
- Respiratory infection (defined as having cough or shortness of breath)

In infants less than 6 months, additionally include those who present with:

- Apnea (temporary cessation of breathing from any cause); and/or
- Sepsis, defined as:
- Fever (37.5 °C or above) or hypothermia (less than 35.5 °C); and
- Shock (defined as lethargy, fast breathing, cold skin, prolonged capillary refill or fast weak pulse); and
- Seriously ill with no apparent cause

In group B subjects not fulfilling the WHO “extended SARI” case definition will also be tested for RSV.

### Exclusion criteria

Neonates younger than 4 days old will not be tested for RSV or influenza due to the high incidence of respiratory symptoms related to intrapartum-related complications or prematurity in this group.

### Sample collection

A nasopharyngeal swab (flocked swab, COPAN, 3 ml universal transport medium (UTM)) will be obtained as soon as possible but no later than 72 h after admission to the PICU and/or HDU. Samples will be taken by trained healthcare staff.

### Point-of-care testing

The samples will be tested for RSV and influenza using the highly sensitive and specific point-of-care (POC) ID NOW test [12–14]. On-site training for local study staff on performing the POC test will be provided by the RSV GOLD team. If site visits are not possible, the training will be given online. Refresher training will be provided before the start of each new respiratory season or if required by the study sites.

### Point-of-care test confirmation

Although the POC ID NOW RSV test has shown high sensitivity and specificity of 100% and 97%, respectively, in previous studies [13], polymerase chain reaction (PCR) remains the gold standard for confirmation of a positive or negative test. POC RSV test confirmation is an optional part of the protocol. Samples will preferably be shipped to the University Medical Centre Utrecht (UMCU) laboratory for conventional or real-time PCR testing in order to confirm the POC RSV test and ensure quality of the data. Researchers may decide to perform viral testing and/or sequencing at a local, national or academic reference laboratory accredited to international quality standards with validated conventional or real-time RSV PCR tests when the capacity is available or when shipment of samples to UMCU is not possible.

Per study site, the following samples will be confirmed:

- All RSV-positive samples.
- A matched (by age and month of admission) number of RSV-negative samples.

### Sample storage

Nasopharyngeal samples will be stored in freezers before shipment. For budget restrictions, influenza-positive samples will not be confirmed through PCR and will not be stored.

### RSV sequencing

RSV sequencing is an optional part of the protocol. Samples sent for confirmation will be stored at the UMCU or study site to allow for sequencing if the sample is RSV-positive. Investigating the molecular heterogeneity of RSV isolates can be important to determine susceptibility or resistance to future RSV monoclonal antibodies or vaccines.

### Data collection and data management

From each study site baseline data will be obtained using questionnaires to evaluate local clinical treatment and management availability and standards. Included patients will be followed up until death or discharge. Participant data will be collected through a case report form and parental questionnaire (Additional file 1: Table S1). These data will be entered by hospital study staff into Castor Electronic Data Capture (EDC) system [15]. Data validation, data analysis and interpretation of the data will be performed by the Utrecht-based study team in collaboration with the site investigators.

### Sample size

#### RSV

The aim is to include all children < 2 years of age meeting the case definition admitted to the PICU and/or HDU at each study site each year during the respiratory season. There is no maximum number of patients each study site needs to recruit. We estimated the following number of inclusions for all study sites (Table 3):

**Table 3:** Estimated number of RSV-positive patients and RSV-related deaths

	RSV-positive patients per study site (N, range)	Total RSV-positive patients (N)	Total RSV-related deaths (N)
Group A	30–120	840	84
Group B	10–30	60	6
Total	NA	900	90

#### Group A

Based on an estimated number of admissions, we expect to recruit 2800 patients across 10 study sites, 100–400 patients at each study site. Assuming 30% of patients who fulfill the WHO case definition “extended SARI” will have a positive RSV test, we expect to capture 840 RSV-positive children, 30–120 children at each study site. Assuming a mortality rate of 10% we expect approximately 3–12 RSV-related deaths at each study site, in total N = 84 deaths. We consider this number sufficient for descriptive purposes.

If 2800 patients will be recruited and 30% RSV-positives are observed (N = 840), this produces a two-sided 95% Clopper-Pearson confidence interval with a width equal to 0.034, ranging from 28 to 32%. In the smallest estimate, 100 patients will be recruited per site. With an assumed RSV-positive sample proportion of 30%, a sample size of 100 patients produces a two-sided 95% confidence interval with a width equal to 0.187, with a lower limit of 21% and an upper limit of 40%.

In 840 RSV-positive patients, an observed proportion of mortality of 10% will produce a two-sided 95% confidence interval with a width equal to 0.042, producing a lower limit of 8.1% and an upper limit of 12.2%. In the smallest estimate, 100 patients will be recruited per site. With an observed mortality proportion of 10%, a sample size of 100 patients produces a two-sided 95% confidence interval with a width equal to 0.127, corresponding to a lower limit of 4.9% and an upper limit of 17.6%. We consider those estimates sufficiently precise.

#### Group B

We expect to recruit 1200 patients in 3 study sites, 200–600 patients at each study site. Assuming 5% of patients will have a positive RSV test, we expect to capture 60 RSV-positive children, 10–30 children at each study site. Assuming a mortality rate of 10% in children who tested positive for RSV, we expect an additional 1–3 RSV-related deaths at each study site, in total N = 6 deaths.

When the sample proportion is 5%, a sample size of 1200 patients produces a two-sided 95% confidence interval with a width equal to 0.026, corresponding to a confidence interval from 3.8 to 6.4%.

In 60 RSV-positive patients, an observed proportion of mortality of 10% will produce a two-sided 95% confidence interval with a width equal to 0.167, with a lower limit of 3.8% and an upper limit of 20.5%.

### Sensitivity WHO case definition

The sensitivity of the WHO “extended SARI” case definition will be calculated using the total population from the 3 sites that included group A and B for RSV testing. In total, we expect to recruit 800 patients from group A and 1200 patients from group B at 3 study sites, in total 2000 patients. We will not adapt the sample size for this purpose as this is a secondary endpoint of the study.

#### Influenza (secondary objective)

Per site we expect to test for influenza in 100–400 (group A) children. Assuming a 5% positivity rate we expect to capture 42 influenza-positive children, 5–20 children at each study site. Assuming a mortality rate of 10%, we expect approximately 1–2 influenza-related deaths at each study site for group A, in total N = 4 deaths.

### Statistical analysis

We will describe characteristics of RSV-positive children. Chi-square tests and nonparametric tests will be used to compare clinical and demographic characteristics between children where appropriate. RSV-positive children from group A and group B will be presented as proportions with 95% confidence intervals. The estimated case fatality ratio in RSV positive children will be provided with 95% confidence intervals. We will also report the mortality rate with 95% confidence intervals, and total RSV-related mortality in group A and B. Subgroup analysis per site will also be performed. We will calculate the sensitivity of the WHO case definition “extended SARI”. We will divide the number of RSV-positive children meeting the case definition (group A) by the total number of RSV-positives regardless of whether the case definition was met and express it as a percentage. No formal statistical analysis plan was written before the start of this descriptive study.

### Burden of disease

The estimated number of RSV-related PICU and/or HDU admissions and deaths in children with respiratory infection in the specific country of participating study sites will be quantified. A numerator (the number of POC-confirmed RSV-related admissions and deaths at the PICU and/or HDU) and denominator (population in the hospital catchment area) will be defined. In case the catchment population is not readily available because the facility is not the only one providing in-patient care to the population, it will be estimated based on reviewing hospital administrative datasets and using a hospital admission survey [16]. In the case of limited administrative data or limited resources to perform a hospital admission survey, burden of RSV disease may be described in terms of the proportion of RSV-related PICU and/or HDU admissions (or deaths) among all PICU and/or HDU admissions with LRTI. Other markers of disease burden will also be reported, such as length of stay, duration of oxygen supplementation, etc.

### Ethical considerations

The study will be conducted according to the principles of the Declaration of Helsinki (version 2013), and local law and regulations. Risks and burdens for study subjects are considered minimal. No other safety issues are expected due to the set-up and nature of the study. No Data Safety Monitoring Board will be appointed and no (Severe) Adverse Events will be reported. Written informed consent will be obtained from each patient-participant by research staff or in accordance with local regulations prior to enrolment in the study.

The intended benefits resulting from this study can be divided into 1) direct benefits and 2) indirect benefits. The primary direct benefit for study participants is timely and proper diagnosis of RSV infection which may result in the prevention of unnecessary or inappropriate use of antibiotics. Secondary direct (patient) benefits consist of:

- The ability to determine that RSV is not the cause of disease and that an alternative diagnosis should be considered in case of a negative test;
- The ability to provide parents of RSV-positive children who have been admitted to PICU and/or HDU or who died with information about the cause of or contribution to death.

Indirect (societal) benefits consist of:

- The ability to provide information on disease burden and target populations to policy makers when a vaccine becomes available;
- Giving medical staff insights into the incidence of RSV/influenza-related admissions and mortality at their hospital;
- Identifying RSV and influenza as important causes of PICU and/or HDU admission and death;
- Capacity building by supplying a reliable POC test to confirm or rule out RSV and influenza as a cause of respiratory infection;
- Capacity building through involving local site investigators in conducting clinical research;
- Increasing overall RSV awareness of hospital staff and parents of young children.

For children from HICs, no direct benefits apply, as RSV testing is part of routine care.

Dual ethical review was performed to ensure that the ethical standards in this study are no less stringent than those applicable in the country of the sponsoring organization. This protocol was therefore submitted for ethics review in The Netherlands as well as to all local (and/or national) research ethics committees.

## Discussion

Although global estimates show a high RSV burden in children from LMICs, individual patient-level data are lacking due to limited availability of RSV testing in these countries. The critical lack of diagnostic capacity hampers the ability to distinguish RSV from other causes of severe respiratory infection in children to justify the need for vaccine introduction in LMICs. Disease awareness is essential to introduce RSV interventions, which will become available in the near future. RSV GOLD is the first global online registry for children younger than 5 years who have died with RSV infection. The first results of the RSV GOLD study have been previously published [2, 17]. The registry was extended after publication (RSV GOLD II) and data collection is still ongoing. In order to increase real-time data collection from LMICs, funding was obtained to establish a network of PICUs in 10 different Gavi-eligible countries (RSV GOLD III).

The RSV GOLD III—ICU Network study is a novel collaboration between RSV GOLD and 10 study teams from 3 different continents, aiming at collecting individual patient-level data of young children with severe RSV infection through POC RSV testing. Data analyses will provide insights into potential risk factors for fatal RSV infection at the PICU and/or HDU and differences between patients from various income settings.

Some challenges remain for this type of study. First, this study will take place mainly in tertiary level facilities in urban areas where access to healthcare is likely better than in rural areas. Results may therefore not be representative of the whole country. Second, the definition of an ICU and HDU may differ from country to country and even within a single healthcare system. For example, in some countries, the capacity to mechanically ventilate differentiates a PICU bed from a HDU bed, while in other countries, a PICU bed may be defined as a bed within a hospital area with a higher patient: nurse ratio. In 2017, the task force of The World Federation of Societies of Intensive and Critical Care Medicine proposed a global definition and stratified ICUs based on the intensity of care provided [18]. For this study, we included both PICUs and HDUs according to the definition of the participating study sites, where the most severely ill children are usually admitted. To characterize differences between participating PICUs and HDUs, we will collect information on the capacity of care, such as the number of available ventilators and attending healthcare staff. Third, due to budgetary constraints, we are unable to extend RSV testing to the regular wards. We will therefore likely miss a proportion of potential study participants in case PICU and/or HDU beds are occupied and children with severe respiratory infection are admitted to the regular wards instead. We will make an effort in collecting information on the number of refusals to estimate the potential impact of this study limitation. Also, influenza testing is limited to 3 study sites. However, this study will provide insights into the characteristics of hospitalized children with severe RSV infection in LMICs including complete granular age distribution data which can be used for modelling studies on the impact of upcoming maternal vaccines and monoclonal antibodies against RSV. Fourth, for some study sites it may be difficult to calculate a catchment population due to the presence of other PICUs in the area. We will collect information on the number of other available PICUs and will adjust for this in our calculations. Based on preliminary data we estimate that 2–4 study sites will not have sufficient data to estimate the catchment population.

Finally, the SARS-CoV2 pandemic may affect the number of respiratory admissions, thus study results may not be representative of regular respiratory seasons in participating countries. Since the duration of the study is 2 years and most of the recruitment will take place in 2022, we do not expect this to be a major limitation.

In summary, this global prospective multicenter study will provide a better understanding of the characteristics and mortality rates of children younger than 2 years who are admitted to the PICU and/or HDU with severe RSV infection in LMICs. These results will not only contribute to knowledge on global disease burden and awareness of RSV, but will also provide valuable information to healthcare policy makers on the impact of future RSV prevention strategies.

## Supplementary Information

**Additional file 1. Table S1. **Patient variables collected.

## Data Availability

Not applicable.

## Abbreviations

RSV: Respiratory Syncytial Virus
LRTI: Lower respiratory tract infection
LMIC: Low- and lower-middle income country
VIS: Vaccine investment strategy
ICU: Intensive care unit
HDU: High-dependency unit
NICU: Neonatal intensive care unit
HIC: High-income country
WHO: World Health Organization
SARI: Severe acute respiratory infection
UTM: Universal transport medium
POC: Point-of-care
PCR: Polymerase chain reaction
UMCU: University Medical Centre Utrecht
EDC: Electronic data capture
