# Correction to: Describing global pediatric RSV disease at intensive care units in GAVI-eligible countries using molecular point-of-care diagnostics: the RSV GOLD-III study protocol

**DOI:** 10.1186/s12879-021-06671-x

**Published:** 2021-09-16

**Authors:** Yvette N. Löwensteyn, Natalie I. Mazur, Harish Nair, Joukje E. Willemsen, Ghislaine van Thiel, Louis Bont, The RSV GOLD III—ICU Network Study Group, Maria Ahuoiza Garba, Fatima Jumai Giwa, Mohammad Hafiz Rasooly, Aminullah Shirpoor, Merwais Azizyar, Lamin Makalo, Ousman Nyan, Ali Mohamed, Khalid Osman, Ram Hari Chapagain, Krishna Prasad Bista, Arun Kumar Sharma, Prabina Shrestha, Bamenla Goka, Kwabena Osman, Evangeline Obodai, Henshaw Mandi, Lucas Esuh Esong, Charlotte Ekoube Eposse, Valéria Muando, Tufária Mussá, Yasser Habresh Said, Aika Abia Shoo, Vanessa Jaelle Dor, Jacqueline Gautier, Lynda Abicher

**Affiliations:** 1 Division of Infectious Diseases, Department of Paediatrics, University Medical Centre Utrecht, Utrecht, The Netherlands; 2 Centre for Global Health, Usher Institute, Edinburgh Medical School, University of Edinburgh, Edinburgh, UK; 3 Respiratory Syncytial Virus Network (ReSViNET) Foundation, Zeist, The Netherlands; 4 Julius Center for Health Sciences and Primary Care, Medical Humanities Department, University Medical Center Utrecht, Utrecht, The Netherlands

## Correction to: BMC Infect Dis (2021) 21:857 https://doi.org/10.1186/s12879-021-06544-3

Following publication of the original article [1], the authors identified an error in the author name of Lynda Abicher.

The incorrect author name is: Linda Abicher.

The correct author name is: Lynda Abicher.

The original article [1] has been corrected.
