# Supplementary material for: Describing global pediatric RSV disease at intensive care units in GAVI-eligible countries using molecular point-of-care diagnostics: the RSV GOLD-III study protocol
Source: BMC Infect Dis. 2021 Aug 23;21:857. doi: 10.1186/s12879-021-06544-3 (PMC8380869; doi:10.1186/s12879-021-06544-3)
Supplement: Supplementary file 1 — Additional file 1. Table S1. Patient variables collected. [file 12879_2021_6544_MOESM1_ESM.docx]

**Additional file 1: Table S1** Patient variables collected

| **Case report form** |  |
| --- | --- |
| **Variables** | **Description** |
| **Age** |  |
| Age at testing | Age at testing in months or days |
| Age at moment of death (if applicable) | Age at moment of death in months or days |
| Date of death (if applicable) | Day - month - year |
| **Basic patient characteristics** |  |
| Sex | Male / Female |
| Severe underlying disease / comorbidity | Congenital heart disease / chronic lung disease / primary/congenital immunodeficiency (not HIV) / genetic/chromosomal disease / Down syndrome / neuromuscular disease / neurodevelopmental disease / airway abnormality / malignancy / malaria / HIV/AIDS / tuberculosis / other / none |
| Length / height at admission | Length or height at admission in cm / inches |
| Weight at admission | Weight at admission in kilograms / pounds |
|  |  |
| Current type of feeding | Exclusive breastfeeding / mixed breast & bottle feeding / exclusive bottle feeding / other / no data available |
| Vitamin D administration | Yes / no / no data available |
| **Perinatal history** |  |
| Prematurity (defined as <37 completed weeks of gestation) | Yes / no / no data available |
| Gestational age | Gestational age in weeks |
| Gestational age estimated by | LMP / ultrasonography / fundal height / unknown |
| Birth weight | Birth weight in kilograms / pounds / no data available |
| Mother immunized during pregnancy | Yes / no / no data available |
| Immunized for | Influenza / pertussis / tetanus / other / unknown |
| Antenatal care visit mother | Yes / no / no data available |
| How many times | Number of antenatal care visits mother |
| **Hospital admission** |  |
| Diagnosis at admission | Admission diagnosis (free text) |
| Referral from other health facility | Yes / no |
| Reason for referral | Referral reason (free text) |
| Referred from | Other hospital / health center / other |
| Date of hospital admission | Day - month - year |
| Date of discharge from hospital | Day - month - year |
| Child was admitted to | HDU / NICU / PICU / emergency ward |
| Length of stay at HDU / NICU / PICU / emergency ward | Length of stay in days |
| Respiratory support | Yes / no |
| Type of respiratory support | Mechanical ventilation / non-invasive ventilatory support (CPAP or high flow nasal canula) / oxygen |
| Duration of respiratory support | Number of days the child received respiratory support |
| Time interval between onset of respiratory infection-related symptoms and first contact with health care provider | Number of days between onset of symptoms and first contact health care provider |
| Time interval between onset of respiratory infection-related symptoms and hospital admission | Number of days between onset of symptoms and hospital admission |
| Location of death (if applicable) | PICU / NICU / HDU / pediatric ward / emergency ward / other / no data available |
| Time interval between onset of respiratory infection-related symptoms and death (if applicable) | Number of days between onset of symptoms and death |
| Referral to another hospital or health facility | Yes / no |
| **Clinical characteristics** |  |
| Clinical symptoms at diagnosis | Cough / difficulty breathing / fast breathing (age <2 months, ≥60 breaths; age 2–11 months, ≥50/min; age 1–5 years, ≥40/min) / chest indrawing / central cyanosis / severe respiratory distress (e.g. grunting, very severe chest indrawing) / inability to breastfeed or drink, vomiting everything / lethargy, reduced level of consciousness or convulsions / fever (temperature >38°C/100°F) / unknown |
| Oxygen saturation (SpO2) at hospital admission | Percentage oxygen saturation at hospital admission / unknown |
| White blood cell (WBC) count at diagnosis | WBC count in cu mm / unknown |
| Haemoglobin (Hb) level at diagnosis | Hb level in g/dl / mmol/l |
| Respiratory samples tested for other pathogens | Yes / no / unknown |
| Respiratory samples tested for other pathogens by | PCR / immunofluorescence / enzyme immune assay / culture / ID NOW influenza A&B / other |
| Other respiratory virus or bacteria present in respiratory sample | Yes / no / unknown |
| Virus or bacteria present in respiratory sample | Influenza A/B / Para-influenza virus / Human metapneumovirus / Adenovirus / Rhinovirus / Streptococcus pneumonia / Haemophilus influenza / Mycoplasma pneumonia / SARS-CoV-2 (COVID-19) / Other |
| Micro-organisms present in other sample | Blood / CSF / urine / other / not measured / no |
| Micro-organisms found in other sample | Free text |
| Pneumothorax | Yes / no / unknown |
| Other clinical diagnoses for this hospital admission | Meningitis / encephalopathy / sepsis / pneumonia/LRTI / other / no / unknown |
| Direct cause of death | Free text |
| Indirect cause of death | Free text |
| **Treatment and prophylaxis** |  |
| Specific RSV care | Yes / no |
| Specific RSV care received | Inhaled bronchodilators / systemic corticosteroids / ribavirin / antibiotics / chest physiotherapy / maintaining hydration and fluid balance / epinephrine / nebulized hypertonic saline / nebulized normal saline / nasal drops normal saline / antitussive, expectorants, decongestants |
| RSV prophylaxis (palivizumab) administered | Yes / no / no data available |
| Immunization status | Received vaccinations according to local immunisation schedule and age / partly vaccinated / not vaccinated / no data available |
| Comments or additional information | Free text |
| **Parental questionnaire** |  |
| **Caregiver** |  |
| Answers to the questionnaire provided by | Mother / father / other family member / other |
| **Questions about the child** |  |
| Does your child have brothers or sisters younger than 12 years living in the same household? | Yes / no / unknown |
| Does your child attend daycare or shared care where other children are present? | Yes / no / unknown |
| Does your child suffer from any underlying diseases? | Free text |
| What type of feeding did your child receive during the first 4 months of age? | Exclusive breastfeeding / mixed breast & bottle feeding / exclusive bottle feeding / other / no data available |
| Does your child receive daily vitamin drops and/or has your child received daily vitamin drops in the past? | Yes / no / unknown |
| Who administers the vitamin drops? | Mother / father / other family member / other |
| Did your child receive vaccinations? | Received all vaccinations according to local immunisation schedule and age / partly vaccinated / unknown |
| **Questions about the pregnancy** |  |
| Did the mother receive antenatal care e.g. see a midwife or doctor before birth of your child? | Yes / no / unknown |
| How many times? | Number of times |
| Was your child born prematurely (<37 weeks of pregnancy)? | Yes / no / unknown |
| Do you know after how many weeks of pregnancy your child was born? | Yes, …. weeks / no / unknown |
| Birthweight of your child | Birthweight in kilograms / pounds |
| Was the mother immunized during her pregnancy? | Yes / no / unknown |
| Immunization | Influenza / pertussis / tetanus / other / unknown |
| **Questions about your hospital visit** |  |
| What symptoms did your child have that made you decide to go to hospital? | Free text |
| How many days after the start of symptoms did you first contact a doctor? | Number of days between onset of symptoms and first contact with a health care provider. |
| How many days after the start of symptoms was your child admitted to the hospital? | Number of days between onset of symptoms and hospital admission. |
| Do you have access to health facilities? | Community health centre / hospital / other / none / unknown |
| What is your travel time to the nearest health facility? | <15 min / 15-60 min / 1-6 hours / >6 hours / unknown |
| What is your travel time to the hospital? | <15 min / 15-60 min / 1-6 hours / >6 hours / unknown |
| What is the distance from home to the hospital? | Km / mile / unknown |
| **Questions about your education and living situation** |  |
| How many years of education has mother completed? | Completed years of education for the mother |
| What is the highest level of education mother has attended? | Uneducated / primary school level / secondary school level / university level / no data available |
| How many years of education has father completed? | Completed years of education for the father |
| What is the highest level of education father has attended? | Uneducated / primary school level / secondary school level / university level / no data available |
| Does your household own or have a TV? | Yes / no / unknown |
| Does your household own or have a refrigerator? | Yes / no / unknown |
| Does your household own or have a phone? | Yes / no / unknown |
| Does your household own or have a bike? | Yes / no / unknown |
| Does your household own or have a car? | Yes / no / unknown |
| Does your household own or have a cheap utensil (<$50): | Yes / no / unknown |
| Does your household own or have an expensive utensil (>$300): | Yes / no / unknown |
| Does your household have electricity? | Yes / no / unknown |
| What is the quality of the main source of drinking water? | Low / middle / high / unknown  (High quality is private piped water or bottled water; middle quality is public tap or standpipe, tube well or borehole, protected well or spring, or tanker truck; low quality is unprotected well or spring, cart with tank/drum, or surface water) |
| What is the quality of the toilet facility usually used? | Low / middle / high / unknown  (High quality is private flush toilet (or flush toilet if private unknown); middle quality is public toilet, ventilated/improved pit latrine, pit latrine with slab, or composting toilet; low quality is pit latrine without slab, open pit, bucket, hanging toilet or no toilet) |
| What is the quality of the main floor material? | Low / middle / high / unknown  (High quality is finished floor with parquet, carpet, tiles, linoleum, ceramic etc.; middle quality is cement, concrete, wood, bamboo etc.; low quality is none, earth, dung etc.) |
| What are the number of rooms used for sleeping? | 1 / 2 / 3/3+ / unknown |
| Does the mother and/or the father smoke? | Yes / no / unknown |
| **Questions about awareness** |  |
| Had you heard about RSV (Respiratory Syncytial Virus) before this hospital admission? | Yes / no / unknown |
| What do you know about RSV (Respiratory Syncytial Virus)? | Free text |
| Had you heard of the influenza virus ("FLU") before this hospital admission? | Yes / no / unknown |
| What do you know about influenza virus? | Free text |
| Had you heard of the rotavirus before this hospital admission? | Yes / no / unknown |
| What do you know about rotavirus? | Free text |
